# Cytokine Profile in Patients with Multiple Sclerosis Following Exercise: A Systematic Review of Randomized Clinical Trials

**DOI:** 10.3390/ijerph19138151

**Published:** 2022-07-02

**Authors:** Parisa Najafi, Maryam Hadizadeh, Jadeera Phaik Geok Cheong, Hamidreza Mohafez, Suhailah Abdullah

**Affiliations:** 1 Centre for Sport and Exercise Sciences, Universiti Malaya, Kuala Lumpur 50603, Malaysia; pari.najafi7452@gmail.com (P.N.); jadeera@um.edu.my (J.P.G.C.); 2 Department of Biomedical Engineering, Faculty of Engineering, Universiti Malaya, Kuala Lumpur 50603, Malaysia; h.mohafez@um.edu.my; 3 Department of Medicine, Faculty of Medicine, Universiti Malaya, Kuala Lumpur 50603, Malaysia; suhailahabd08@yahoo.com

**Keywords:** multiple sclerosis, inflammatory markers, demyelinating autoimmune diseases, exercise, rehabilitation, cytokine, interleukins, tumor necrosis factor alpha

## Abstract

Multiple sclerosis (MS) is one of the most prevalent causes of nontraumatic neurological impairment in young adults. This review aims to determine the impact of exercise on cytokine and adipokine profile levels as inflammatory markers in MS patients across various exercise paradigms. We used specific keywords in PubMed, Web of Science, The Cochrane Library, and Scopus to find randomized clinical trials addressing the effects of physical activity and exercise training on inflammatory markers levels in MS patients. The majority of the research showed no considerable changes in IL-6 levels, while three studies reported declining levels after the intervention. Approximately half of the trials observed a change in TNF-α and IL-10 levels after exercise interventions, while the other half showed no meaningful changes. Other markers such as IL-17, IL-4, IL-12, adipokines, and BDNF showed fluctuations in levels. We found no universal agreement on the effects of different exercise training protocols on the serum level of inflammatory markers in patients with MS. More research is needed to fully identify the effects of exercise on cytokines in MS patients.

## 1. Introduction

Multiple sclerosis (MS) is defined as an immune-mediated inflammatory, neurodegenerative, and demyelinating disease that impacts the central nervous system (CNS) in young individuals [1,2]. Approximately 1 to 2.5 million people (mostly women, with a female-to-male ratio of 2:1) around the world are affected by MS [3]. A variety of genetic and environmental factors, including immune system dysregulation, central nerve demyelination [4,5], vitamin D deficiency, Epstein–Barr virus, and smoking [6], have been considered as possible etiologies of MS. Ultimately, the exact primary nature of MS pathogenesis remains unknown [7,8]. Although there are controversies regarding the exact mechanism of disease initiation, it is well known that inflammation plays an important role in pathogenesis of the disease [9]. Inflammation promotes neurodegeneration and demyelination, starting with plaque formation in the white matter and progressing to macrophage and T-cell aggregation in the peripheral blood circulation [10,11]. As important players in inflammation, cytokines [12] are proteins that are released from the bloodstream, the cerebrospinal fluid (CSF), or both, which modulate the maturation and function of immune cells [13,14].

In contrast to healthy individuals, over-secretion of T helper 1 (Th1) and proinflammatory cytokines leads to imbalanced serum levels of tumor necrosis factor (TNF-α), interferon-gamma (IFN-γ), interleukin (IL)-1, IL-2, IL-12, IL-15, IL-6, chemokine (C–X–C motif) ligand 8 (CXCL8) and CXCL13, chemokine (C–C motif) ligand 20 (CCL20), T helper 2 (Th2) and anti-inflammatory cytokines IL-4, IL-5, IL-10, and IL-13 in MS patients [7,15]. Adipokines such as leptin, a proinflammatory cytokine, and adiponectin, an anti-inflammatory mediator, are cytokines produced by adipose tissue, playing crucial roles in the progression and pathogenesis of MS [16]. In addition, secretion of proinflammatory factors is increased and secretion of anti-inflammatory cytokines is decreased in MS, which may result in intensified demyelination [7]. Recent studies demonstrated that CXCL8, TNF-α, IL-12p40, IL-15, and CXCL13 are enhanced in both CSF and blood in MS patients. Although TNF-α, IL-12, IL-13, CCL-5, CXCL12, CXCL13, IFN-γ, IL-12p70, and CXCL8 were all increased in a moderate way, CCL20, IL-23, IL-21, IL-12p40, IL-17F, IL-22, and IL-2R had a larger effect in comparison to other cytokines in persons with multiple sclerosis (PwMS) [7]. Thus, maintaining the balance between Th1 and anti-inflammatory cytokines is associated with control of the MS pathogenic process, including axonal demyelination [17]. Previous studies considered a modifying effect of exercise activity on adipokines and cytokines with potential control over MS progression [16,18,19,20]. Researchers accurately documented the cytokine response to exercise training in healthy people which varies in different modes, intensity, and duration. However, there were some discrepancies between similar studies in patients with MS [17,21,22].

Moderate exercise had a positive impact on low-grade inflammatory markers such as IL-6 and leptin [22,23]. Exercise led to increased anti-inflammatory markers in the initial response, but a decrease was also reported following a period of regular activity [21,24,25]. Conversely, there have been no reported significant changes in IL-17, TNF-α, IFN-γ, and IL-10 after exercise [11,16,26,27]. Two studies indicated that drawing clear conclusions about the impact of training on cytokine (ILs and TNF-α) and adipokine (leptin) levels in MS patients is impossible, and that exercise had no effect on MS clinical manifestations of systemic inflammation [11,17].

The effects of post-exercise cytokines and adipokines on MS patients are still in the early stages of research, and results are in doubt. Due to the above differences in the effect of exercise on cytokines and adipokines, this study was undertaken to comprehensively evaluate randomized clinical trials (RCTs) examining the effects of exercise on inflammatory markers that have shown moderate to large changes in PwMS. In the hopes of acquiring insight into the effects of cytokines and adipokines on the pathogenesis of MS, as well as the role of exercise, finding an alternative complementary treatment for these patients is a priority. In addition, the present research provides a systematic review of the effect of exercise on PwMS as a secondary aim of mental and physical health.

## 2. Materials and Methods

### 2.1. Guideline

The PRISMA 2020 guidelines [28] were used to conduct this systematic review. Our research was submitted to PROSPERO (the International Prospective Register of Systematic Reviews) with the following number: CRD42021272187 (https://www.crd.york.ac.uk/prospero/display_record.php?ID=CRD42021272187) (accessed on 6 August 2021). The literature search started prior to registration, and data extraction was in progress (but not completed) when we registered.

### 2.2. Literature Search Strategy

A search was independently conducted through electronic databases including Scopus, Web of Science, The Cochrane Library, and PubMed by two researchers to find studies addressing the effects of physical activity and/or exercise training on serum levels of inflammatory markers in PwMS. The search language was restricted to English and Persian. The search date was limited to studies that were published from January 2003 to April 2022. This systematic review was conducted using specific keywords such as “multiple sclerosis” AND (“exercise” OR “yoga” OR “physical endurance” OR “exercise movement techniques” OR “resistance training”) AND (“interleukins” OR “tumor necrosis factor-alpha” OR “cytokines” OR “inflammation” OR “interferons” OR “adipokines” OR “leptin” OR “chemokines”). The two reviewers conducted the searches independently and prescreened the initial stage of the study selection, including the analysis of titles and abstracts. In the second stage, the full-text studies were evaluated to select them according to the eligibility criteria. A third author was responsible for supervising the procedure and resolving any discrepancies. Reference lists of eligible publications and similar systematic reviews were checked to find any potential studies. We used EndNoteX20 (Malaysia, Kuala Lumpur, UM Library-PTM) issued by Thomson Reulers as a tool to manage references.

### 2.3. Eligibility Criteria

**Figure 1:**
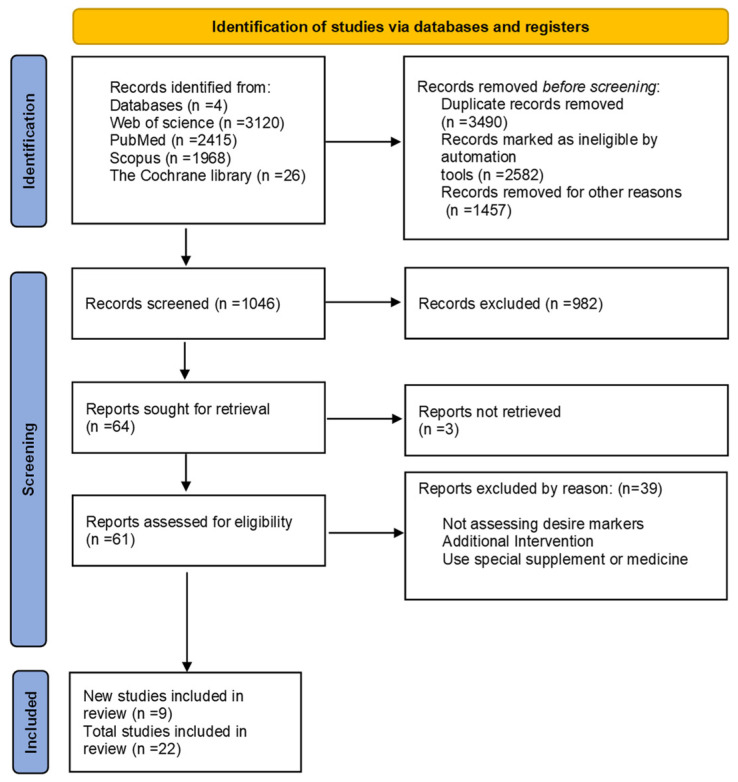
PRISMA flow diagram study selection and inclusion process.

All RCTs that evaluated the effect of any exercise or physical activity on the serum level of inflammatory markers in MS patients were included. Cytokines, chemokines, and adipokines were among the inflammatory markers evaluated in the blood and CSF in this research. Studies included had to satisfy the following criteria: (1) RCTs if they were well described and of high-quality, with defined outcomes; (2) studies on MS patients who engage in regular physical activity; (3) research on proinflammatory and anti-inflammatory cytokines or adipokines. Moreover, studies with the following criteria were excluded from the current review: articles not in English or Persian, nonhuman trials, interventions besides supplements and medicines, absence of full-text studies, duplicate reports, reviews, studies, comments, opinion pieces, methodological reports, and conference abstracts. Figure 1 depicts the screening process.

### 2.4. Study Selection and Data Extraction

Data from the studies were independently collected and recorded in a Microsoft Excel database by two reviewers. Again, these processes were supervised by a third researcher. Variables extracted included the first author’s name, gender, sample size, age, disease status, Expanded Disability Status Scale (EDSS), type of exercise, duration and frequency of exercise, evaluated cytokines, a secondary outcome, type of sampling, and final results of each paper.

### 2.5. Assessment of Methodological Quality

To assess the quality of selected papers, two independent reviewers were asked to use standardized critical assessment instruments from the Joanna Briggs Institute (JBI) System for unified methodological quality of papers selected [29]. Each study was given a score between 0 and 13 points. Q1. Was true randomization used for assignment of participants to treatment groups? Q2. Was allocation to treatment groups concealed? Q3. Were treatment groups similar at the baseline? Q4. Were participants blind to treatment assignment? Q5. Were those delivering treatment blind to treatment assignment? Q6. Were outcomes assessors blind to treatment assignment? Q7. Were treatment groups treated identically other than the intervention of interest? Q8. Was follow-up completed and, if not, were differences between groups in terms of their follow-up adequately described and analyzed? Q9. Were participants analyzed in the groups to which they were randomized? Q10. Were outcomes measured in the same way for treatment groups? Q11. Were outcomes measured in a reliable way? Q12. Was appropriate statistical analysis used? Q13. Was the trial design appropriate, and any deviations from the standard RCT design (individual randomization and parallel groups) accounted for in the conduct and analysis of the trial? The RoB tool’s items were scored as “yes” (low risk of bias), “no” (high risk of bias), or “unclear or unapplicable” (indicating that the item was not reported and, as a result, the possibility of bias was unknown). All manuscript sections were screened for report quality. Table 1 presents the supporting information.

## 3. Results

In total, 7529 papers were found; 3490 duplicates and 4039 articles that did not fulfill the inclusion criteria were removed, leaving 1046 papers for abstract and title screening. Following a full-text review, 22 articles were chosen for further analysis. The PRISMA flowchart shows a summary of the search and research selection process (Figure 1).

### 3.1. Characteristics of the Eligible Studies

The included studies were RCTs investigating the effects of any exercise on the serum level of inflammatory markers in the blood and CSF in MS patients. The characteristics of these studies and the changes in inflammatory markers are summarized in Table 2, while Table 3 shows the secondary outcome, which includes mental and physical components. Most of the studies were conducted in Asia and Europe (91%), while two studies (9%) were conducted in Latin America and the USA. About 419 and 323 individuals of both genders participated in the exercise and control groups, respectively. Thirteen studies evaluated both male and female patients. Eight studies were conducted on female subjects, and only one study investigated male participants. Patients with relapsing–remitting multiple sclerosis (RRMS) were assessed in 13 studies. While three and four studies included primary progressive multiple sclerosis (PPMS) and secondary progressive multiple sclerosis (SPMS) patients, respectively, the others did not specify the type of MS. In the included studies, the EDSS of the patients ranged from 0 to higher than 6.5. Furthermore, only one study examined cytokines in both CSF and blood [23], while others used blood serum levels. A majority of trials excluded participants with a history of taking immunomodulatory medicines for 24 h to 6 months prior to the intervention.

### 3.2. Study Quality and Risk of Bias

All of the articles were RCTs according to the definition used by the JBI’s critical assessment tool [29]. Twenty-two publications met 9–11 criteria, which were considered of high quality, and none of the studies were excluded. All studies were well designed and carried out. The JBI RoB results are elaborated in Table 1.

### 3.3. Classification of Evidence

#### 3.3.1. IL-6

Interleukin-6 was the most commonly assessed inflammatory marker, which was reported in 14 out of 22 studies [18,19,20,24,26,27,30,31,32,33,34,35,36,37]. The majority of the research showed no considerable changes in IL-6 levels, including five studies after using ergometers [19,20,26,34,36], two studies with combined training [18,33], one study after aerobic training [27], and another study with resistance exercise [35]. Three studies reported a decrease after 8 and 12 weeks (t.i.w.) of combined training [30,37] and 12 weeks of resistance exercise (b.i.w.) [32]. Only two studies evaluated high levels of IL-6 after one session of fitness [24] and 8 weeks (b.i.w.) of cycle ergometer exercises [31].

#### 3.3.2. TNF-α

TNF-α was studied in 11 of 22 trials [16,18,19,26,27,33,34,35,38,39,40], with the majority of studies showing no significant difference in TNF-α levels with varying exercises from one session for 24 weeks [19,27,33,35,38,39]; 8 weeks of aerobic (t.i.w.) exercise [16], 12 weeks (t.i.w.) of combined training [18], and one session of ergometer training [26] resulted in lower serum TNF-α levels. Only one study reported an increase in serum TNF-α level following 8 weeks (t.i.w.) of cycle ergometer exercises [34]. 

#### 3.3.3. IFN-γ

Eight studies reported IFN-γ serum levels; however, three studies reported no significant changes after three treadmill sessions [27], 12 weeks (t.i.w.) of combined exercise [18], and 24 weeks (b.i.w.) of resistance exercises [38]. Moreover, three studies evaluated a lower level of IFN-γ after 8 weeks of combined training [39,40,41] and 8 weeks of resistance exercises [35]. In contrast, in two other studies, IFN-γ was increased after 8 weeks of ergometer exercises [34] and 12 weeks of combined exercise [30].

#### 3.3.4. IL-12 and IL-12p70

Two studies reported a decrease in post-exercise serum level (8 to 12 weeks, 2 to 3 days a week) following resistance and aqua training, respectively [32,42]. Only one study reported no significant changes after combined training [33], while another reported no significant changes after 8 weeks of ergometer exercises in serum levels of IL-12p70 [31].

#### 3.3.5. IL-10

Eleven trials assessed IL-10 levels, and no noticeable changes in IL-10 levels were detected after performing combined exercises in three studies [18,33,39], and after resistance [38], Pilates [43], and aerobic exercises [16] in one study each. Although, four studies reported a significant reduction in IL-10 level after resistance training [35], ergometer exercises [26,31], and three sessions of aerobic exercises [27], one research showed an enhanced IL-10 level after 8 weeks (t.i.w.) of combination training [37].

#### 3.3.6. IL-4

Two trials found no significant differences after 8 weeks (t.i.w.) of combined training [41], three treadmill sessions [27], and 24 weeks of resistance exercise [38]; White et al. and Kierkegaard et al. found a decline in serum IL-4 levels after 8 and 12 weeks (b.i.w.) resistance training, respectively. 

#### 3.3.7. Adipokines

Three trials examined leptin following exercise, and two of them also assessed adiponectin. Two of them reported a decline in leptin serum levels after one session on an ergometer [26] and 8 weeks of aerobic training [16]. Only Ebrahimi et al. reported no considerable difference after 10 weeks (t.i.w.) of WBV. Furthermore, two studies following aerobic training [16] and one session of ergometer training [26] observed an increase and no changes in adiponectin serum levels, respectively. 

#### 3.3.8. BDNF

Three studies from five trials [19,20,24,36,43] reported a boost in BDNF serum levels after 3 and 9 (b & t.iw.) weeks of cycle ergometry [19,20], and 8 weeks of Pilates training [43]. BDNF serum levels remained unchanged in two studies with ergometer exercises and fitness interventions [24,36].

### 3.4. Physical and Mental as a Secondary Outcome

Functional muscle strength [23,32,35,36,38,41] and fatigue [16,19,23,32,35,36] were the most examined factors, as evidenced by six articles, the majority of which showed better muscle function, and half of which reported fatigue treatments. Five trials reported an improvement in QoL and walking function after the period of intervention [23,26,30,31,32,36,38].

## 4. Discussion

There is scarce literature assessing the effects of exercise on inflammatory markers in PwMS with introduction of exercise as a complementary therapy for modifying cytokine and adipokine secretions. Although the majority of trials showed fluctuations in the secretion of cytokines and adipokines, none of them indicated that exercise causes intensification and progression of disease in MS patients. These fluctuations may be associated with methodological flaws. On the other hand, MS is considered an inflammatory autoimmune disease for which the fundamental cause has remained unknown, after decades.

### 4.1. Proinflammatory Cytokines

Interleukin-6 is a myokine released when skeletal muscle contracts, and nine studies reported no change in IL-6 levels after various exercises [12,18,19,20,27,34,35,36,44]. The current review is in agreement with the findings of a previous large review [17], which reported that seven trials from eight studies did not show a statistically significant difference in IL-6 levels after exercise. A possible explanation for the observed lack of IL-6 decrease in response to exercise may be that baseline IL-6 levels are not elevated in MS patients, as would be expected with MS pathogenesis as an inflammatory disease. This hypothesis is supported by a recent systematic review, which assessed 48 articles showing that IL-6 levels in the blood and CSF of MS patients were not elevated as compared to healthy individuals [7]. It is also suggested that MS patients with high IL-6 levels be included before designing a new study to examine the effect of exercise. Moreover, because exercise causes the release of IL-6 in skeletal muscles, peritendinous tissues, and the brain [27], it may be useful for future studies to also examine IL-6 levels in the blood, skeletal muscles, and CSF after intervention. In contrast to earlier findings [17], three studies with moderate intensity and frequency demonstrated a decrease in cytokine levels after exercise [30,32,37].

TNF-α is another important proinflammatory cytokine, which was studied in 11 trials. Although this study supports evidence from previous observations [17], the majority of studies found no significant changes in TNF-α following exercise training [16,26,27,33,35,38,39], while four trials indicated lower serum levels. According to the review by Bai et al., TNF- α levels in CSF and blood are higher in MS patients. Meanwhile, the reduction in Th1 after exercise is also due to a boost in cortisol and adrenaline levels in response to physical activity, which results in a decline in TNF-α produced by Th1 cells [44,45]. Only one study observed an increase in TNF-α [34], which supports the idea of a cytokine boost in serum levels via an increase in their receptors after exercise [11,17]. The scarcity and weakness of the research, the fluctuation of TNF-α levels after exercise, and the complex role of TNF-α make it difficult to fully interpret the findings. Prior investigations recommended that increased TNF-α in the blood may have a helpful [46] or harmful [47] impact on PwMS. For example, while an increase in TNF-α in CSF and blood may be associated with the stage of blood–brain barrier dysfunction [47], it may also be associated with mild drops in disease relapses [46]. All of these contradictory findings reveal that the method of training, the timing, and the type of sampling (CSF or blood) may all have an impact on the variations of cytokine production [11,17].

IFN-γ is released by T cells and natural killer cells, which are not naturally found in the CNS. According to a recent review [7], IFN-γ levels are moderately increased in MS patients when compared to healthy individuals. Although eight studies in our review showed greater fluctuation after interventions [18,27,30,34,35,39,41], our findings support evidence from previous observations showing that IFN-γ is frequently reduced by prolonged and intense activity [11,48]. This decrease implies that physical activity can naturally decline the number of peripheral blood Th-1 cells and their ability to secrete the proinflammatory cytokine. Despite these discrepancies, our findings indicate that exercise can decline serum interferon levels and the role of IFN-γ after exercise. Nevertheless, additional research into the effects of exercise is required, taking into account the type and intensity, frequency, and duration of exercise, as well as gender.

Another proinflammatory cytokine that plays an important role in the immunopathology is IL-17, which is found in the CSF and blood of PwMS patients, with a large increase during relapses [49]. Contrary to previous expectations [11], this study showed a decrease in IL-17 serum levels, which had a beneficial impact on the amount of inflammation in PwMS patients who were in the stable/remission phase and were relapse-free for at least 2 months. Moreover, this could be related to the flow of the protocol in the intervention method. The IL-12 family has also been reported to have moderate blood and CSF levels in PwMS. However, there has been less discussion about these two cytokines in the previous literature [11,17], and our results did not show consistent changes in serum levels. As such, this information was not enough to provide a definitive assessment of the factors affecting IL-12 family levels.

### 4.2. Anti-Inflammatory Cytokines

IL-10 is an anti-inflammatory cytokine of the Th2 type that causes disease improvement, remission, and recovery periods in PwMS [37]. It was the third most evaluated cytokines among studies [16,18,26,27,31,33,35,37,38,39,43], with five studies finding no significant changes. Although these findings support the outcomes of previous reviews [11,17], the majority of trials showed no statistically significant changes. This outcome was reflected by Bai et al. (2019), who evaluated 24 studies and did not reveal a difference in blood serum levels of IL-10 among PwMS and healthy people. Thus, in forthcoming research, more conclusive evidence may be needed to examine IL-10 following exercise. Furthermore, as IL-6 secretion decreases, IL-10 as an anti-inflammatory marker and TNF-α as a proinflammatory marker increase and decrease, respectively [38,43], which can manage and improve MS pathogenic functions such as axonal transection and demyelination [17]. Additionally, the assessment of anti-inflammatory markers such as IL-10 is worthless without considering proinflammatory markers such as IL-6 and TNF-α after exercise. The role of IL-4 in the pathogenesis of MS has been less commonly discussed in the literature. It was evaluated in five studies, and no considerable differences were reported in cytokine levels [27,38,41]. These results corroborate the findings of many previous studies [11,17] that showed ambiguity results regarding the effect of exercise on IL-4.

### 4.3. Adipokines

Adipokines are cytokines that manage and drive the production of proinflammatory cytokines such as TNF-α, as well as boost inflammatory signals and plague development. Leptin was one of the first adipokines to be discovered, and adiponectin is an anti-inflammatory agent [45]. Our findings reflect the results of Negaresh et al. (2018), who discovered a link between adipokine alternation and fat mass, intensity, and exercise protocol. In general, our study and previous studies [11,17] did not present adequate results to suggest that exercise programs are useful in modifying adipokine levels in PwMS.

### 4.4. BDNF

Brain-derived neurotrophic factor is a CNS protein which improves mood or cognition in PwMS [24], where it was evaluated alongside other inflammatory markers. Szuhany et al. (2015) reported that regular exercise, albeit at a low intensity, can increase the level of BDNF in PwMS. In addition, it was shown that the benefits of these changes may be lower in women than in men. However, our findings do not support previous research [50]; the limited RCTs evaluated in this study were not adequate to interpret the results.

### 4.5. Physical and Mental Health as a Secondary Outcome

In 10 studies, mental and physical factors and inflammatory markers were measured simultaneously [16,23,25,30,31,32,35,36,38,41]. A few trials indicated a rise and decline in the concurrent accumulation of anti- and proinflammatory markers and an individual’s physical and mental functions. This finding broadly supports a systematic review [11] which indicated that an increase or decrease in anti- and proinflammatory markers was not necessarily correlated with an improvement in mental and physical factors after exercise. On the other hand, these findings may be because of the limited experimental trials performed to ascertain this correlation between inflammatory markers and mental and physical health among PwMS. There is abundant room for further progress in determining the correlation of physical and mental factors with a wider range of inflammatory markers after exercise. Furthermore, using different methodologies, such as a large sample size and a long duration, may yield different results for future research. As such, exercise may be introduced as a complementary therapy that can improve physical and mental function in PwMS.

### 4.6. Future Study

According to the results of the present review and similar articles, so far, the focus has been on evaluating inflammatory markers such as IL-6 and IL-10 (which are nonsignificant); thus, future studies should focus on the factors that cause the greatest changes in the blood and CSF in MS patients, including TNF-α, CXCL8, IL-15, IL-12p40, and CXCL1 [7]. In addition, these discrepancies in the results may be due to the weakness of the research method, which sometimes used one gender or did not have a control group or healthy control group in the study, which should be addressed in future trials. Furthermore, all the studies that were assessed in the current study implemented only a single type of intervention, and they did not provide any comparison between various types of training. As such, it is suggested that future studies on inflammatory markers be designed to compare two different types of exercise with the same duration and frequency. To improve quality, future studies should include high-quality, large-scale, standardized RCTs that define the appropriate and standardized method, exercise choices, intensity, and duration of intervention. This will help to limit the risk of bias. More attention should also be given to the type and protocol of exercises. Moreover, obese adipose tissue releases visfatin, TNF-α, IL-6, leptin, angiotensin II, and resistin [51], among which the leptin level is significantly related to subcutaneous fat expression and BMI [23]. Furthermore, weight loss during exercise may alter the profile of cytokines and adipokines such as leptin, and more research is needed to completely understand which protocol of exercise is promising. Moreover, only one study evaluated the cytokine level in CSF and blood. Further work is required to assess the cytokine and adipokine levels in CSF and other tissues such as skeletal muscles and fat following intervention. According to the previous literature, inflammation was shown to increase in the early stages of MS and the relapsing–remitting phase. As such, considerably more work will need to be conducted to determine the effects of physical exercise at an early stage and in the relapsing–remitting phase on inflammatory markers. Moreover, inflammation can diminish quality of life by degrading otherwise healthy parts of the body. Therefore, it is important to design studies to assess the effects of exercise on MS patient population according to the different degrees of inflammation. The present studies were designed to evaluate the limited inflammatory markers after physical activity. On the other hand, this would be a fruitful area for further trials to assess protein markers, such as complement proteins and proteins involved in neural development and maintenance in PwMS after exercise. Although single-cell immune profiling has received more attention in the pathogenesis of MS, the complementary role of proteomics should not be ignored. Lastly, it is recommended that studies based on single-cell proteomics be conducted in MS patients following exercise.

### 4.7. Strengths and Limitations

The relatively large number of trials included was the current review’s main strength. We were able to pool data from 22 RCTs, whereas previous systematic reviews obtained data from fewer trials. Another significant strength of our review was that it evaluated RCTs until 2022, whereas the last review only considered studies up until 2017. The search strategy of the present article was more comprehensive and broader than that used in previous research [17]. Nonetheless, there were some limitations to this study.

Firstly, despite the notable prevalence of MS, most of the included studies had a limited number of patients, which is another factor affecting generalization and causing experimental bias. Secondly, we could not perform a meta-analysis due to the heterogenicity of exercise protocols and their duration, as well as the remarkable differences in study populations, in terms of both gender distribution and disease type or severity (EDSS: 0–8).

## 5. Conclusions

The current review did not provide a consensus on the effects of different exercise training protocols on the serum level of inflammatory markers in patients with MS. This may be attributed to variations in the population gender, design and duration of studies, and inflammatory marker measurement protocols. Although it was indicated that acute exercise induces short-term inflammation that followed by a mid-term anti-inflammatory environment, there are still many unanswered questions about the beneficial methodological flaws in the face of inflammatory factors.

## Figures and Tables

**Table 1:** JBI tool for assessing RCTs.

JBI Critical Appraisal Checklist for Randomized Controlled Trials														
Study	Q1	Q2	Q3	Q4	Q5	Q6	Q7	Q8	Q9	Q10	Q11	Q12	Q13	Total
Tadayon Zadeh F.	Y	Y	Y	NA	NA	U	Y	Y	Y	Y	Y	Y	Y	10
Devasahayam A.	Y	Y	Y	NA	NA	U	Y	Y	Y	Y	Y	Y	Y	10
Faramarzi M.	Y	Y	Y	NA	NA	U	Y	Y	Y	Y	Y	Y	Y	10
Rezaee S.	Y	Y	Y	NA	NA	U	Y	Y	Y	Y	Y	Y	N	9
Nejatpour S.	Y	Y	Y	NA	NA	U	Y	Y	Y	Y	Y	Y	Y	10
Barry A.	Y	Y	Y	NA	NA	Y	Y	Y	Y	Y	Y	Y	N	10
Berkowitz S.	Y	Y	Y	NA	NA	U	Y	Y	Y	Y	Y	Y	N	9
Eftekhari E.	Y	Y	Y	NA	NA	U	Y	Y	Y	Y	Y	Y	Y	10
Majidnasab N.	Y	Y	Y	NA	NA	U	Y	Y	Y	Y	Y	Y	Y	10
Mokhtarzade M.	Y	Y	Y	NA	NA	U	Y	Y	Y	Y	Y	Y	Y	10
Alvarenga-Filho H.	Y	Y	Y	NA	NA	U	Y	Y	Y	Y	Y	Y	Y	10
Briken S.	Y	Y	Y	NA	NA	U	Y	Y	Y	Y	Y	Y	Y	10
Kierkegaard M.	Y	Y	Y	NA	NA	U	Y	Y	Y	Y	Y	Y	Y	10
Deckx N.	Y	Y	Y	NA	NA	Y	Y	Y	Y	Y	Y	Y	Y	11
Ebrahimi A.	Y	Y	Y	NA	NA	U	Y	Y	Y	Y	Y	Y	Y	10
Kjølhede T.	Y	Y	Y	NA	NA	U	Y	Y	Y	Y	Y	Y	Y	10
Bansi J.	Y	Y	Y	NA	NA	U	Y	Y	Y	Y	Y	Y	N	9
Golzari Z.	Y	Y	Y	NA	NA	U	Y	Y	Y	Y	Y	Y	Y	10
Castellano V.	Y	Y	Y	NA	NA	U	Y	Y	Y	Y	Y	Y	Y	10
White L.J.	Y	Y	Y	NA	NA	U	Y	Y	Y	Y	Y	Y	N	9
Schulz K.	Y	Y	Y	NA	NA	U	Y	Y	Y	Y	Y	Y	Y	10
Heesen C.	Y	Y	Y	NA	NA	U	Y	Y	Y	Y	Y	Y	Y	10

Y, yes; N, no; NA, not applicable; U, unclear. Critical appraisal criteria for quantitative studies: Q1. Was true randomization used for assignment of participants to treatment groups? Q2. Was allocation to treatment groups concealed? Q3. Were treatment groups similar at the baseline? Q4. Were participants blind to treatment assignment? Q5. Were those delivering treatment blind to treatment assignment? Q6. Were outcomes assessors blind to treatment assignment? Q7. Were treatment groups treated identically other than the intervention of interest? Q8. Was follow-up completed and, if not, were differences between groups in terms of their follow-up adequately described and analyzed? Q9. Were participants analyzed in the groups to which they were randomized? Q10. Were outcomes measured in the same way for treatment groups? Q11. Were outcomes measured in a reliable way? Q12. Was appropriate statistical analysis used? Q13. Was the trial design appropriate, and any deviations from the standard RCT design (individual randomization and parallel groups) accounted for in the conduct and analysis of the trial?

**Table 2:** Sample characteristics and main findings of the reviewed studies.

First Author	Gender	Sample Size	Mean Age	Disease Status	Mean EDSS	Type of Exercise	Duration and Frequency of Exercise	Evaluated Cytokines	Main Findings
Tadayon Zadeh F.	Female	MST:15 MSC:15	Range (25–40)	-	≤6	Endurance, resistance, balance	8 wks (t.i.w.), 40–70% HR max	IL-6, CRP, IL-10	↓: IL-6, CRP ↑: IL-10
Devasahayam A.	Both	MST:14 MSC:8	54.07 (8.46)	SPMS PPMS	6–6.5	Fitness	One session	BDNF, IL-6	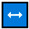 : BDNF ↑: IL-6
Faramarzi M.	Female	MST:46 MSC:43	Range (18–50)	RRMS	Not reported range (0–8)	Combined stretching, balance, pilates, resistance, endurance	12 wks (t.i.w.)	IL-6, IFN-γ, CRP	↓:IL-6, CRP ↑: IFN-γ
Rezaee S.	Both	MST:10 MSC:10	28.9 ± 3.3	RRMS	2.2 ± 0.4	Aerobic training	6 wks (t.i.w.) 60% VO_2_ max	TNF- α	↓: TNF- α
Nejatpour S.	Male	MST:13 MSC:12	-	-	Range (2.5 –5)	Aqua training	8 wks (t.i.w.) 75% VO_2_ max	IL-12, Il-17	↓:IL-12, IL-17
Barry A.	Both	MST:9 HC:10	35.33 ± 2.12	RRMS	2.17 ± 0.40	Cycle ergometer	8 wks (b.i.w.), 65–75% VO_2_ max	IL-10, IL-12p70, IL-6	↑: IL-6, 12 p70 ↓: IL-10
Berkowitz S.	Female	MST:15 MSC:10	33.8 ± 7.8	-	1.5	Aerobic (treadmill) 50–80 VO_2_ max	3 sessions	IL-4, IL-6, IL-10, IL-17A, IFN-γ, TNF-α	↓: IL-10 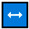 : IL-4, IL-17A, IFN-γ, TNF-α, IL-6
Eftekhari E.	Female	MST:15 MSC:15	34.46 ± 7.29	RRMS	Range (2–6)	Pilates training	8 wks (t.i.w.)	IL-10, BDNF	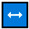 : IL-10 ↑: BDNF
Majidnasab N.	Female	MST:30 HC:15 MSC:5	28.23 ± 3.65	RRMS	2.11 ± 0.76	Arm, cycle ergometer	One session 60–70% VO_2_ max	IL-6, IL-10, TNF- α, leptin, adiponectin	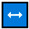 : IL-6, adiponectin ↓: TNF- α, IL-10, leptin
Mokhtarzade M.	Female	MST:22 MSC:8	32.04 ± 2.81	RRMS	1.84 ± 0.35	Aerobic	8 wks (t.i.w.) 60% max watt	IL-10 TNF- α Leptin adiponectin	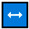 : IL-10 ↓: leptin, TNF- α ↑: adiponectin
Alvarenga-Filho H.	Both	MST:8 MSC:10 HC:10	41.1 ± 12.9	RRMS	0–2.5	Resistance training, cycle ergometer, pilates	12 wks (t.i.w.)	IL-6, IL-10, IL-21, IL-22, IL-17, TNF-α, IFN-γ	↓:IL-22 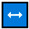 : IL-17, -10, -21, TNF- α, IL-6, IFN-γ
Briken S.	Both	MST:28 MSC:9	49.9 ± 7.6	PPMS, SPMS	4.9 ± 0.9	Endurance, arm ergometer, cycle ergometer, rowing	9 wks (b & t.i.w.)	BDNF, IL-6	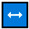 : IL-6 ↑: BDNF
Kierkegaard M.	Both	MST:20	36.3 ± 7.6	RRMS	1.5	Resistance training	12 wks (b.i.w.) 80% 1 RM	IL-1ra, -4, -5, -6, -7, -8, -12p70, -13, -17	↓: all in blood 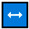 : all in CSF
Deckx N.	Both	MST:29 MSC:16	47 ± 2	RRMS and CPMS	3 ± 0.2	Endurance, resistance	12 wks (t.i.w.)	IL-6, IL-10, IL-12p70, TNF- α	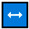 : all
Ebrahimi A.	Both	MST:16 MSC:14	38.76 ± 9.66	RRMS	3.11 ± 0.99	WBV	10 wks (t.iw.)	leptin	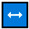 : leptin
Kjølhede T.	Both	MST:16 MSC:16	44.6 ± 7	RRMS	2.9 ± 1	Progressive resistance training	24 wks (b.i.w.)	IL-1β, IL-4, IL-10, IL-17F, IL-23, TNF-α, IFN-γ	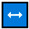 : all
Bansi J.	Both	WT:24 LT:28	44.6–56.3	-	4.65	Cycle ergometer, aquatic bike	3 wks, 70% Hpeak	BDNF, TNF- α, IL-6, sIL-6r	↑: BDNF 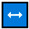 : NGF, TNF-α, IL-6, sIL-6r
Golzari Z.	Female	MST:10 MSC:10	32.15 ± 7.57	-	2.14 ± 1.06	Stretch, aerobic, resistance, endurance	8 wks (t.i.w.)	IFN-γ, IL-4, IL-17	↓: IFN-γ, IL-17 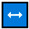 : IL-4
Castellano V.	Both	MST:11 MSC:11	40 ± 10	-	0–5.5	Cycle ergometer	8 wks (t.i.w.) 60% VO_2_ max	TNF- α, IL-6, IFN-γ	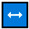 : IL-6 ↑: TNF- α, IFN-γ
White L.J.	Female	MST:11	47 ± 12	-	3.8 ± 0.9	Resistance training	8 wks (b.i.w.) 50–70% MVC	IL-2, IL-4, IL-6, IL-10, TNF-α, IFN-γ, CRP	↓:IL-4, IL-10, IFN-γ, IL-2, CRP 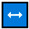 : IL-6, TNF- α
Schulz K.	Both	MST:15 MSC:13	42 ± 9.5	RRMS, SPMS	2.5 ± 1.4	Cycle ergometer	8 wks (b.i.w.) 75% VO_2_ max	BDNF, NGF, IL-6, sIL-6r	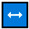 : All
Heesen C.	Both	MST:15 MSC:13 HC:20	39.8	RRMS, SPMS, PPMS	2.3 ± 0.2	Cycle ergometer (resistance + endurance)	8 wks (b.i.w.) 60% VO_2_ max	IFN-γ, TNF- α, IL-10	↓: IFN-γ 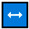 : TNF- α, IL-10

Weeks = wks; three times a week= (t.i.w.); two times a week = (b.i.w.); two and three times a week= (b & t.i.w.), not reported = (-), MS = multiple sclerosis; IL = interleukin; TNF-α = tumor necrosis factor alpha; RCT = randomized controlled trial; RRMS = relapsing–remitting multiple sclerosis; PPMS = primary progressive multiple sclerosis; CNS: central nervous system; SPMS = secondary progressive multiple sclerosis; CPMS = chronic progressive multiple sclerosis; BDNF = brain-derived neurotrophic factor; NGF = nerve growth factor; CRP = C-reactive protein; IFN-ɣ = interferon-γ; EDSS = Expanded Disability Status Scale; IL-1Ra = IL-1 receptor antagonist; Th = T-helper; ↓:increased; ↑:decreased; 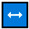: no significant changes; MST: MS training group; MSC: MS control group; HC: healthy control group; WT: water training group; LT: land training group.

**Table 3:** The values of physical and mental indices in MS individuals after intervention.

Study	Sample Type	Functional and Mental Outcomes	Results
Tadayon Zadeh F.	Blood	-	-
Devasahayam A.	Blood	-	
Faramarzi M.	Blood	Walking function	Improvement in walking function
Rezaee S.	Blood	-	-
Nejatpour S.	Blood	-	-
Barry A.	Blood	QoL Depression	Improvement in QoL and depression
Berkowitz S.	Blood	-	-
Eftekhari E.	Blood	-	-
Majidnasab N.	Blood	-	-
Mokhtarzade M.	Blood	Fatigue QoL	Improvement in fatigue and QoL
Alvarenga-Filho H.	Blood	-	-
Briken S.	Blood	-	-
Kierkegaard M.	Blood and CSF	QoL Mood Muscle strength function Walking function Cognition Fatigue	Improvement in QoL, mood, muscle strength function, walking function, cognition, and fatigue
Deckx N.	Blood	-	-
Ebrahimi A.	Blood	Fatigue QoL Balance Walking function Muscle strength function	Improvement in balance and walking function No significant changes in QoL, muscle strength function, and fatigue
Kjølhede T.	Blood	Muscle strength Walking function	Improvement in muscle strength function and walking function
Bansi J.		Fatigue	No significant changes
Golzari Z.	Blood	Balance Muscle strength function	Improvement in balance and muscle strength function
Castellano V.	Blood	-	-
White L.J.	Blood	Muscle strength function Fatigue	Improvement in muscle strength function and fatigue
Schulz K.	Blood	QoL Fatigue Muscle strength function Walking function	Improvement in QoL No significant changes in fatigue, muscle strength function, and walking function
Heesen C.	Blood	-	-

QoL: quality of life.

## Data Availability

Data are available on request from the authors.
